# Newspaper depiction of mental and physical health in Qatar

**DOI:** 10.1192/bji.2020.11

**Published:** 2021-02

**Authors:** Khalid Elzamzamy, Abdulkarim Alsiddiqi, Ali Khalil, Hassan Elamin, Mustafa Abdul Karim, Ovais Wadoo

**Affiliations:** 1 MD, Psychiatry Resident, Psychiatry Department, Hamad Medical Corporation, Doha, Qatar. Email: Kh.zamzamy@gmail.com; 2 MD, Psychiatry Resident, Psychiatry Department, Hamad Medical Corporation, Doha, Qatar; 3 MBBS, MSc, MRCPsych, CCT, Consultant Psychiatrist, Psychiatry Department, Hamad Medical Corporation, Doha, Qatar

**Keywords:** Newspapers, Qatar, media coverage, mental health and illness, physical health

## Abstract

This study provides an overview of the extent, nature and quality of reporting on mental health compared with physical health in Qatari newspapers. We analysed 1274 news reports from daily newspapers in Qatar. The majority of the articles provided general information and were either positive or neutral in tone, reporting purely on physical health matters. A small proportion made associations with violence or reported on suicide or substance use. Our results highlight the underrepresentation of mental health in Qatari newspapers. A collaboration between media and health professionals is recommended to improve reporting on mental health.

Qatar is a rapidly developing high-income Middle Eastern country with a total population of 2.7 million.^1^ Qatar's official language is Arabic and its official religion is Islam.

Qatar's healthcare system is predominantly state-funded. Primary Health Care Corporation provides primary healthcare services through country-wide health centres. Hamad Medical Corporation is the main provider of secondary and tertiary healthcare, with growing numbers of specialised services, including mental health services.

The prevalence of psychiatric disorders in Qatar is comparable to international figures. Anxiety and depressive disorders are the most common psychiatric disorders.^2^ Mental health has been recognised by the state as a public health priority, as evidenced by the two consecutive versions of the National Mental Health Strategy for 2013–2018 and 2018–2022. One of the main objectives of the strategy is to increase public awareness and reduce the stigma associated with psychiatric disorders.

Global research findings indicate that media portrayal of psychiatric disorders shapes public attitudes and may contribute to the associated stigma. In other words, media not only reflects but creates public attitudes towards mental health. Therefore, numerous global studies analysed the media's depiction of mental illnesses.^3^ However, there is not much data about Arab media. McCrae et al recently studied media portrayal of psychiatric disorders in Saudi Arabia,^4^ but there are no similar studies from Qatar and thus an important research gap exists in this field.

Our study generally aimed at providing an overview of the extent, nature and quality of portrayal of mental health compared with physical health in Qatari newspapers.

## Method

Seven daily newspapers in Qatar were surveyed over a period of 2 months between 1 April and 31 May 2018. The newspaper selection included four Arabic (*Al-Sharq*, *Al-Watan*, *Al-Raya*, *Al-Arab*) and three English (*The Peninsula*, *Gulf Times*, *Qatar Tribune*) dailies. This study included only national newspapers, as they are the most widely distributed. Electronic (PDF) versions of the newspapers were obtained from their respective websites. Newspapers were manually screened for headlines about any health-related topics. All health-related articles were included in our study.

**Fig. 1:**
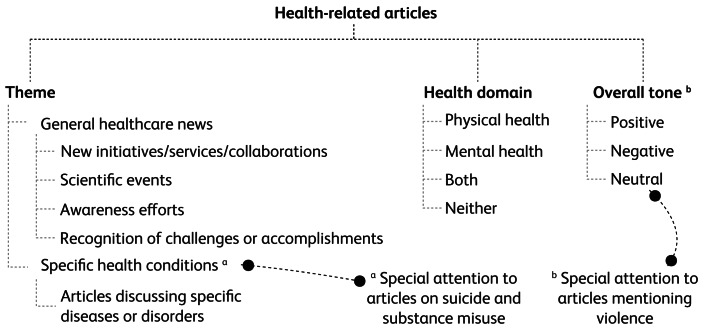
System of categorisation of the studied articles, based on three domains.

The main body of the included articles was read, analysed and categorised on the basis of three main domains: theme; health domain; and the overall tone of the article (Fig. 1). According to the theme of the news conveyed in the articles, they were classified into (a) general and (b) specific articles. General articles were further classified into one of four domains: new initiatives/services/collaborations; scientific events; awareness efforts; and recognition of challenges or accomplishments. Specific articles are those that addressed specific health conditions, diseases or disorders. Special attention was given to articles on suicide, in order to compare them with international guidelines on suicide reporting. Substance misuse was another topic that we paid special attention to because of the reported magnitude of the problem in Qatar.

Within the health domain, each article was classified into physical health, mental health, both or neither. Articles were also classified, on the basis of their overall tone, into positive, negative or neutral (Box 1). Special attention was paid to articles linking health and illness with violence.

Box 1 Examples of headlines with positive, negative and neutral tone

**Positive**

Articles that invoke a sense of hope, trust, care and understanding:

‘HMC [Hamad Medical Corporation] stresses on patient safety and quality of care’
‘Ministry asks private clinics to cut charges’
‘HMC wins a prestigious award’
‘Qatari nurses encouraged to develop skills and knowledge’

**Negative**

Articles that highlight shortcomings, system challenges, deficiencies, risks, negative outcomes, or make negative associations (e.g. with violence):

‘HMC services are not meeting the needs of the society’
‘Delay in outpatient appointments: a chronic problem’
‘Online medication prescriptions: a danger that is one click away’
‘The emergency department needs emergency help’

**Neutral**

Mainly articles stating facts:

‘Avoiding red meat may protect against cancer’
‘5.1 million outpatient visits each year’
‘A new affiliation between Hamad University and Al-Shafallah Center’
‘A seminar on vitamin D’

The necessary ethical approvals to conduct this study were obtained from the Medical Research Centre at Hamad Medical Corporation, Qatar.

## Results

Our screening of headlines yielded 1274 health-related news articles published during the 2 months. The majority of the articles (60.8%) were on purely physical health topics, and only 16.6% reported on mental health. Some articles (7.7%) had a mixed content covering both physical and mental health elements, and 14.9% were not directly related to either (Table 1).

**Table 1:** Comparison between physical and mental health reporting in seven Qatari daily newspapers, 1 April to 31 May 2018

Variable	Physical health		Mental health		Both		Neither		Total	
	*n*	%	*n*	%	*n*	%	*n*	%	*n*	%
Articles identified	775	60.8%	211	16.6%	98	7.7%	190	14.9%	1274	100%
Overall tone										
Positive	306	39.5%	98	46.4%	54	55.1%	76	40%	534	41.9%
Negative	104	13.4%	29	13.7%	8	8.2%	25	13.2%	166	13%
Neutral	365	47.1%	84	39.8%	36	36.7%	89	46.8%	574	45.1%
Theme										
General healthcare news	442	57%	119	56.3%	65	66.3%	182	95.8%	808	63.4%
Specific health conditions	333	43%	92	43.6%	33	33.6%	8	4.2%	466	36.5%
Special topics										
Association with violence									6	0.5%
Substance misuse									9	0.7%
Suicide									20	1.6%

Most articles were either positive or neutral in tone (41.9 and 45.1% respectively), with only 13% of articles negatively portraying health-related issues, with equal distribution between mental and physical health. Minor differences were noted between the tone of physical and mental health articles (Table 1). Overall, a small proportion made associations with violence (0.5%), all of which were related to mental health.

Although almost one-third of the articles addressed specific physical or mental conditions/disorders, the majority of the articles (63.4%) provided general healthcare information with no specific focus on a condition or disorder. From the totality of articles, 1.6% focused only on suicide and less than 1% focused on substance use problems.

## Discussion

The stigma associated with psychiatric disorders has been construed as a social phenomenon rooted in social relationships and shaped by the culture and structure of society.^5^ Psychiatric disorders are among the most stigmatised conditions in both Western and Eastern cultures. The role of media in reinforcing or challenging the stigma is paramount. In Qatar, the media is considered a primary source of information about mental health issues^6^ and, therefore, the information provided by the media is central to shaping the public's knowledge and attitudes.

The results of our study indicate that reporting on physical health was almost three times more frequent than reporting on mental health. International studies show similar underreporting on mental health.^7,8^ This limited coverage could move mental health further down the public agenda and miss opportunities to counter the stigma associated with psychiatric disorders.

The results of our study also indicate that the portrayal of mental health was much more positive than is typical in newspapers from other countries around the world. Analyses of global media have shown extensive associations between mental disorders, violence and crime.^7^ This leads to the perception that individuals with psychiatric disorders are violent. A recent study conducted in Saudi Arabia showed that one-third of the articles included in the analysis carried the theme of ‘dangerousness’.^4^

In our study, 1.6% of the articles were on suicide. There are no data available to see whether there was an increase in the number of suicides following the reporting in Qatar. A large body of research shows a link between media portrayal of suicide and subsequent increase in suicide attempts and ‘copycat suicide’.^9^ Suicide is a sensitive issue – it is illegal in Qatar and this could be the reason for underreporting. One retrospective study found that 470 individuals presented to an emergency department in Doha over the course of 1 year (from July 2011 to July 2012) with completed suicide (48), suicide attempts (165), intentional self-harm (105) and accidental self-harm (152).^10^ In our sample, reporting on suicide was at times irresponsible and not in keeping with the international guidelines on suicide reporting. For example, some of the articles used language that sensationalised, normalised or presented suicide as a solution to problems. Other articles did not avoid pictures or explicit description of the method used in the suicide. Suicide articles did not provide information about where to seek help.

In our study, less than 1% of articles reported on alcohol and substance misuse. This is a very low figure when compared with studies from other countries.^11^ It has been reported in the media that up to 5% of Qatar's population aged 18–60 years has substance use problems related to alcohol and other drugs.^12^

In our study, there was a preponderance of general news articles announcing new initiatives, services, collaborations, and scientific and awareness events. This reflects the developing nature of Qatar and the huge investments made by the government in developing world-class healthcare services and scientific institutes.

It is pertinent that Qatar is one of only four countries in the world that currently have partnerships with The Carter Centre in the USA to train journalists as part of the Rosalynn Carter Fellowships for Mental Health Journalism. Qatari fellows have been joining the fellowship since 2016. Our study did not analyse the contributions of the Carter Mental Health Journalism Fellows to the reporting on mental health in Qatar.

### Recommendations

Although our study found that mental health is underrepresented in Qatari newspapers compared with physical health, it is encouraging that, overall, they report sensitively and positively on mental health issues. A collaboration between media and health professionals in Qatar would be critical to improving the quality of reporting on mental health, perhaps through developing culturally sensitive reporting guidelines. Future studies may look at the contributions of the Carter Journalism Fellows to mental health reporting.

### Limitations

One of the limitations of our study was the exclusion of other media sources, such as broadcast media, and also international newspapers distributed in Qatar. Thus, our findings cannot be generalised beyond local newspapers. Our results represent the articles published within a limited period (2 months). A sample of longer duration may have improved the generalisability of the results. The categorisation of the tone of the articles was to some extent subjective and relied on each reviewer's impression of the report. Measures to ensure consistency between reviewers included pre-review orientation, clear examples of categories and discussion of uncertain cases. Formal testing of interrater reliability was not performed in this study.
